# (*E*)-Methyl 3-(1*H*-indol-3-yl)acrylate

**DOI:** 10.1107/S1600536811046745

**Published:** 2011-11-12

**Authors:** Dong-Feng Li, Xiao-Fei Zhu, Shuang Guan, Rui-Bin Hou

**Affiliations:** a School of Chemistry and Life Science, Changchun University of Technology, Changchun 130012, People’s Republic of China

## Abstract

In the title compound, C_12_H_11_NO_2_, the indole and methyl acrylate mean planes are inclined at an angle of 10.6 (1)°. In the crystal, N—H⋯π inter­actions link mol­ecules into chains along [010] and weak inter­molecular C—H⋯O hydrogen bonds further consolidate the crystal packing.

## Related literature

For general background to the synthesis of 3-substituted indole derivatives as precursors of potent anti-inflammatory and analgesic agents, see Radwan *et al.* (1997 ▶). For details of the synthesis, see García-Rubia *et al.* (2010 ▶). For related structures, see: Bhella *et al.* (2009 ▶); Hou & Li (2011 ▶).
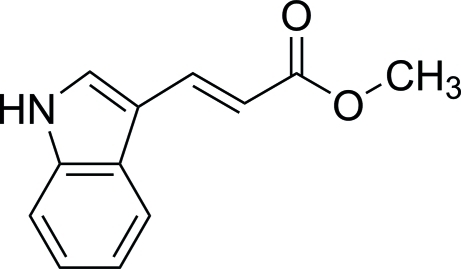


## Experimental

### 

#### Crystal data

C_12_H_11_NO_2_
*M* *_r_* = 201.22
Monoclinic, 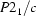

*a* = 5.884 (3) Å
*b* = 7.923 (5) Å
*c* = 21.898 (13) Å
β = 93.54 (3)°
*V* = 1018.9 (10) Å^3^
*Z* = 4
Mo *K*α radiation
μ = 0.09 mm^−1^
*T* = 288 K
0.43 × 0.26 × 0.22 mm

#### Data collection

Rigaku R-AXIS RAPID diffractometer
Absorption correction: multi-scan (*ABSCOR*; Higashi, 1995 ▶) *T*_min_ = 0.963, *T*_max_ = 0.980
9616 measured reflections
2333 independent reflections
1730 reflections with *I* > 2σ(*I*)
*R*_int_ = 0.031

#### Refinement

*R*[*F* ^2^ > 2σ(*F* ^2^)] = 0.042
*wR*(*F* ^2^) = 0.136
*S* = 1.08
2333 reflections
138 parameters
H-atom parameters constrained
Δρ_max_ = 0.20 e Å^−3^
Δρ_min_ = −0.18 e Å^−3^

### 

Data collection: *RAPID-AUTO* (Rigaku, 1998 ▶); cell refinement: *RAPID-AUTO*; data reduction: *CrystalStructure* (Rigaku/MSC, 2002 ▶); program(s) used to solve structure: *SHELXS97* (Sheldrick, 2008 ▶); program(s) used to refine structure: *SHELXL97* (Sheldrick, 2008 ▶); molecular graphics: *SHELXTL* (Sheldrick, 2008 ▶); software used to prepare material for publication: *SHELXL97*.

## Supplementary Material

Crystal structure: contains datablock(s) global, I. DOI: 10.1107/S1600536811046745/cv5189sup1.cif

Structure factors: contains datablock(s) I. DOI: 10.1107/S1600536811046745/cv5189Isup2.hkl

Supplementary material file. DOI: 10.1107/S1600536811046745/cv5189Isup3.cml

Additional supplementary materials:  crystallographic information; 3D view; checkCIF report

## Figures and Tables

**Table 1:** Hydrogen-bond geometry (Å, °) *Cg* is the centroid of the C1–C6 ring.

*D*—H⋯*A*	*D*—H	H⋯*A*	*D*⋯*A*	*D*—H⋯*A*
C8—H8⋯O1^i^	0.93	2.65	3.558 (2)	165
C12—H12*B*⋯O1^ii^	0.96	2.63	3.540 (3)	159
N1—H1*A*⋯*Cg*^iii^	0.86	2.52	3.189 (3)	135

Symmetry codes: (i) 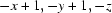; (ii) 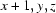; (iii) 
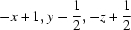
.

**Fig. 1.**
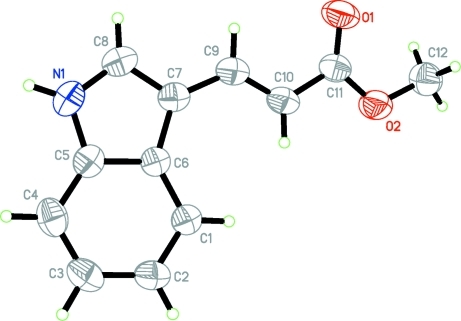
The molecular structure of the title compound showing the atomic numbering and 50% probability displacemnet ellipsoids.

## supplementary crystallographic information

### Comment

Indole skeleton is a key component of many biologically active compounds. Radwan *et al.* (1997) have synthesized and evaluated of 3-substituted indole derivatives as precursors of potent anti-inflammatory and analgesic agents. Recently, Bhella *et al.* (2009) reported a series of compounds with the similar structures. In this paper, we report the crystal structure of the title compound.

In the tiltle compound (Fig. 1), all bond lengths and angles are normal and comparable with those reported for close structures (Bhella *et al.*, 2009; Hou & Li, 2011). The dihedral angle between the indole and methyl acrylate mean planes is 10.6 (1)°. In the crystal structure, N—H···π interactions (Table 1) link molecules into chians along [010], and weak intermolecular C—H···O hydrogen bonds (Table 1) consolidate further the crystal packing.

### Experimental

The title compound was prepared according to the literature (García-Rubia *et al.*, 2010). Single crystals suitable for X-ray diffraction were prepared by slow evaporation a mixture of dichloromethane and petroleum (60–90 °C) at room temperature.

### Refinement

C-bound H atoms were placed in calculated positions (C—H 0.93 and 0.96 Å) and were included in the refinement in the riding model with *U*_iso_(H) = 1.2 and 1.5 *U*_eq_(C). The N-bound H atom was placed in calculated position with N—H = 0.86 Å and refined with *U*_iso_(H) = 1.2 *U*_eq_(N).

### Crystal data

**Table:**

C_12_H_11_NO_2_	*F*(000) = 424
*M**_r_* = 201.22	*D*_x_ = 1.312 Mg m^−^^3^
Monoclinic, *P*2_1_/*c*	Mo *K*α radiation, λ = 0.71073 Å
Hall symbol: -P 2ybc	Cell parameters from 6974 reflections
*a* = 5.884 (3) Å	θ = 3.2–27.5°
*b* = 7.923 (5) Å	µ = 0.09 mm^−^^1^
*c* = 21.898 (13) Å	*T* = 288 K
β = 93.54 (3)°	Block, colourless
*V* = 1018.9 (10) Å^3^	0.43 × 0.26 × 0.22 mm
*Z* = 4	

### Data collection

**Table:**

Rigaku R-AXIS RAPID diffractometer	2333 independent reflections
Radiation source: fine-focus sealed tube	1730 reflections with *I* > 2σ(*I*)
graphite	*R*_int_ = 0.031
ω scans	θ_max_ = 27.5°, θ_min_ = 3.2°
Absorption correction: multi-scan (*ABSCOR*; Higashi, 1995)	*h* = −6→7
*T*_min_ = 0.963, *T*_max_ = 0.980	*k* = −10→10
9616 measured reflections	*l* = −28→28

### Refinement

**Table:**

Refinement on *F*^2^	Secondary atom site location: difference Fourier map
Least-squares matrix: full	Hydrogen site location: inferred from neighbouring sites
*R*[*F*^2^ > 2σ(*F*^2^)] = 0.042	H-atom parameters constrained
*wR*(*F*^2^) = 0.136	*w* = 1/[σ^2^(*F*_o_^2^) + (0.0781*P*)^2^ + 0.0624*P*] where *P* = (*F*_o_^2^ + 2*F*_c_^2^)/3
*S* = 1.08	(Δ/σ)_max_ < 0.001
2333 reflections	Δρ_max_ = 0.20 e Å^−^^3^
138 parameters	Δρ_min_ = −0.18 e Å^−^^3^
0 restraints	Extinction correction: *SHELXL97* (Sheldrick, 2008), Fc^*^=kFc[1+0.001xFc^2^λ^3^/sin(2θ)]^-1/4^
Primary atom site location: structure-invariant direct methods	Extinction coefficient: 0.026 (5)

### Special details

**Table:**

Experimental. (See detailed section in the paper)
Geometry. All e.s.d.'s (except the e.s.d. in the dihedral angle between two l.s. planes) are estimated using the full covariance matrix. The cell e.s.d.'s are taken into account individually in the estimation of e.s.d.'s in distances, angles and torsion angles; correlations between e.s.d.'s in cell parameters are only used when they are defined by crystal symmetry. An approximate (isotropic) treatment of cell e.s.d.'s is used for estimating e.s.d.'s involving l.s. planes.
Refinement. Refinement of *F*^2^ against ALL reflections. The weighted *R*-factor *wR* and goodness of fit *S* are based on *F*^2^, conventional *R*-factors *R* are based on *F*, with *F* set to zero for negative *F*^2^. The threshold expression of *F*^2^ > σ(*F*^2^) is used only for calculating *R*-factors(gt) *etc*. and is not relevant to the choice of reflections for refinement. *R*-factors based on *F*^2^ are statistically about twice as large as those based on *F*, and *R*- factors based on ALL data will be even larger.

### Fractional atomic coordinates and isotropic or equivalent isotropic displacement parameters (Å^2^)

**Table:**

	*x*	*y*	*z*	*U*_iso_*/*U*_eq_	
C1	0.9472 (2)	0.64568 (18)	0.23712 (6)	0.0434 (3)	
H1	1.0528	0.7010	0.2143	0.052*	
C2	0.9795 (3)	0.63514 (19)	0.30016 (7)	0.0502 (4)	
H2	1.1092	0.6829	0.3195	0.060*	
C3	0.8210 (3)	0.55410 (19)	0.33566 (7)	0.0534 (4)	
H3	0.8461	0.5510	0.3780	0.064*	
C4	0.6291 (3)	0.47926 (19)	0.30863 (7)	0.0524 (4)	
H4	0.5245	0.4244	0.3319	0.063*	
C5	0.5973 (2)	0.48890 (17)	0.24505 (7)	0.0434 (3)	
C6	0.7520 (2)	0.57135 (16)	0.20809 (6)	0.0379 (3)	
C7	0.6584 (2)	0.55697 (17)	0.14540 (6)	0.0428 (3)	
C8	0.4580 (2)	0.46741 (19)	0.14818 (7)	0.0503 (4)	
H8	0.3616	0.4387	0.1145	0.060*	
C9	0.7440 (3)	0.61468 (18)	0.08871 (7)	0.0461 (4)	
H9	0.6473	0.6000	0.0539	0.055*	
C10	0.9444 (3)	0.68641 (19)	0.07991 (6)	0.0474 (4)	
H10	1.0461	0.7067	0.1133	0.057*	
C11	1.0071 (3)	0.73389 (19)	0.01826 (6)	0.0462 (4)	
C12	1.3035 (3)	0.8419 (3)	−0.03879 (8)	0.0621 (5)	
H12A	1.2504	0.7638	−0.0700	0.093*	
H12B	1.4669	0.8430	−0.0359	0.093*	
H12C	1.2477	0.9528	−0.0490	0.093*	
N1	0.4222 (2)	0.42746 (16)	0.20675 (6)	0.0520 (4)	
H1A	0.3071	0.3719	0.2184	0.062*	
O1	0.8851 (2)	0.72446 (18)	−0.02800 (5)	0.0711 (4)	
O2	1.22147 (19)	0.79075 (16)	0.01910 (5)	0.0607 (3)	

### Atomic displacement parameters (Å^2^)

**Table:**

	*U*^11^	*U*^22^	*U*^33^	*U*^12^	*U*^13^	*U*^23^
C1	0.0429 (7)	0.0419 (7)	0.0452 (8)	0.0007 (6)	0.0015 (6)	−0.0008 (6)
C2	0.0524 (8)	0.0507 (8)	0.0463 (8)	0.0032 (7)	−0.0077 (7)	−0.0025 (6)
C3	0.0702 (10)	0.0491 (8)	0.0404 (7)	0.0101 (7)	−0.0006 (7)	0.0023 (6)
C4	0.0644 (10)	0.0426 (7)	0.0517 (8)	0.0047 (7)	0.0149 (7)	0.0057 (6)
C5	0.0441 (7)	0.0340 (7)	0.0521 (8)	0.0027 (6)	0.0037 (6)	−0.0018 (6)
C6	0.0396 (7)	0.0316 (6)	0.0426 (7)	0.0043 (5)	0.0018 (6)	−0.0013 (5)
C7	0.0439 (7)	0.0387 (7)	0.0452 (7)	0.0032 (6)	−0.0012 (6)	−0.0043 (6)
C8	0.0449 (8)	0.0497 (8)	0.0553 (9)	−0.0006 (6)	−0.0049 (7)	−0.0077 (7)
C9	0.0496 (8)	0.0454 (8)	0.0423 (7)	0.0048 (6)	−0.0055 (6)	−0.0036 (6)
C10	0.0535 (8)	0.0499 (8)	0.0379 (7)	0.0033 (7)	−0.0037 (6)	−0.0035 (6)
C11	0.0471 (8)	0.0499 (8)	0.0412 (7)	0.0063 (6)	0.0001 (6)	−0.0032 (6)
C12	0.0568 (9)	0.0772 (11)	0.0535 (9)	0.0000 (9)	0.0116 (8)	0.0016 (8)
N1	0.0453 (7)	0.0484 (7)	0.0625 (8)	−0.0086 (5)	0.0057 (6)	−0.0025 (6)
O1	0.0614 (8)	0.1069 (10)	0.0435 (6)	−0.0047 (7)	−0.0078 (5)	0.0081 (6)
O2	0.0550 (7)	0.0826 (8)	0.0444 (6)	−0.0088 (6)	0.0013 (5)	−0.0001 (5)

### Geometric parameters (Å, °)

**Table:**

C1—C2	1.384 (2)	C8—N1	1.350 (2)
C1—C6	1.407 (2)	C8—H8	0.9300
C1—H1	0.9300	C9—C10	1.334 (2)
C2—C3	1.406 (2)	C9—H9	0.9300
C2—H2	0.9300	C10—C11	1.470 (2)
C3—C4	1.376 (2)	C10—H10	0.9300
C3—H3	0.9300	C11—O1	1.2075 (18)
C4—C5	1.396 (2)	C11—O2	1.338 (2)
C4—H4	0.9300	C12—O2	1.442 (2)
C5—N1	1.377 (2)	C12—H12A	0.9600
C5—C6	1.415 (2)	C12—H12B	0.9600
C6—C7	1.452 (2)	C12—H12C	0.9600
C7—C8	1.380 (2)	N1—H1A	0.8600
C7—C9	1.443 (2)		
			
C2—C1—C6	118.88 (14)	N1—C8—H8	124.9
C2—C1—H1	120.6	C7—C8—H8	124.9
C6—C1—H1	120.6	C10—C9—C7	128.21 (14)
C1—C2—C3	121.62 (14)	C10—C9—H9	115.9
C1—C2—H2	119.2	C7—C9—H9	115.9
C3—C2—H2	119.2	C9—C10—C11	121.07 (13)
C4—C3—C2	120.92 (15)	C9—C10—H10	119.5
C4—C3—H3	119.5	C11—C10—H10	119.5
C2—C3—H3	119.5	O1—C11—O2	122.92 (15)
C3—C4—C5	117.46 (15)	O1—C11—C10	125.77 (16)
C3—C4—H4	121.3	O2—C11—C10	111.31 (12)
C5—C4—H4	121.3	O2—C12—H12A	109.5
N1—C5—C4	129.60 (14)	O2—C12—H12B	109.5
N1—C5—C6	107.38 (14)	H12A—C12—H12B	109.5
C4—C5—C6	123.02 (14)	O2—C12—H12C	109.5
C1—C6—C5	118.10 (13)	H12A—C12—H12C	109.5
C1—C6—C7	135.37 (13)	H12B—C12—H12C	109.5
C5—C6—C7	106.53 (12)	C8—N1—C5	109.94 (13)
C8—C7—C9	123.06 (13)	C8—N1—H1A	125.0
C8—C7—C6	105.90 (13)	C5—N1—H1A	125.0
C9—C7—C6	131.02 (13)	C11—O2—C12	116.66 (12)
N1—C8—C7	110.25 (13)		

### Hydrogen-bond geometry (Å, °)

**Table:**

Cg is the centroid of the C1–C6 ring.

**Table:**

*D*—H···*A*	*D*—H	H···*A*	*D*···*A*	*D*—H···*A*
C8—H8···O1^i^	0.93	2.65	3.558 (2)	165.
C12—H12B···O1^ii^	0.96	2.63	3.540 (3)	159.
N1—H1A···Cg^iii^	0.86	2.52	3.189 (3)	135.

Symmetry codes: (i) −*x*+1, −*y*+1, −*z*; (ii) *x*+1, *y*, *z*; (iii) −*x*+1, *y*−1/2, −*z*+1/2.
